# Environmental cold exposure increases blood flow and affects pain sensitivity in the knee joints of CFA-induced arthritic mice in a TRPA1-dependent manner

**DOI:** 10.1186/s13075-015-0905-x

**Published:** 2016-01-11

**Authors:** Elizabeth S. Fernandes, Fiona A. Russell, Khadija M. Alawi, Claire Sand, Lihuan Liang, Robin Salamon, Jennifer V. Bodkin, Aisah A. Aubdool, Matthew Arno, Clive Gentry, Sarah-Jane Smillie, Stuart Bevan, Julie E. Keeble, Marzia Malcangio, Susan D. Brain

**Affiliations:** Centre for Cardiovascular Excellence, Waterloo Campus, King’s College London, Franklin-Wilkins Building, 150 Stamford St, London, SE1 9NH UK; Genomics Centre, Waterloo Campus, King’s College London, Franklin-Wilkins Building, 150 Stamford St, London, SE1 9NH UK; Institute of Pharmaceutical Science, Waterloo Campus, King’s College London, Franklin-Wilkins Building, 150 Stamford St, London, SE1 9NH UK; Programa de Pós-Graduação, Universidade Ceuma, São Luís, MA 65075-120 Brazil; Wolfson Centre for Age-Related Diseases, Guy’s Campus, King’s College London, London, SE1 1UL UK

**Keywords:** Rheumatoid arthritis, Cold, TRPA1, Blood flow, Pain

## Abstract

**Background:**

The effect of cold temperature on arthritis symptoms is unclear. The aim of this study was to investigate how environmental cold affects pain and blood flow in mono-arthritic mice, and examine a role for transient receptor potential ankyrin 1 (TRPA1), a ligand-gated cation channel that can act as a cold sensor.

**Methods:**

Mono-arthritis was induced by unilateral intra-articular injection of complete Freund’s adjuvant (CFA) in CD1 mice, and in mice either lacking TRPA1 (TRPA1 KO) or respective wildtypes (WT). Two weeks later, nociception and joint blood flow were measured following exposure to 10 °C (1 h) or room temperature (RT). Primary mechanical hyperalgesia in the knee was measured by pressure application apparatus; secondary mechanical hyperalgesia by automated von Frey system; thermal hyperalgesia by Hargreaves technique, and weight bearing by the incapacitance test. Joint blood flow was recorded by full-field laser perfusion imager (FLPI) and using clearance of ^99m^Technetium. Blood flow was assessed after pretreatment with antagonists of either TRPA1 (HC-030031), substance P neurokinin 1 (NK_1_) receptors (SR140333) or calcitonin gene-related peptide (CGRP) (CGRP_8–37_). TRPA1, TAC-1 and CGRP mRNA levels were examined in dorsal root ganglia, synovial membrane and patellar cartilage samples.

**Results:**

Cold exposure caused bilateral primary mechanical hyperalgesia 2 weeks after CFA injection, in a TRPA1-dependent manner. In animals maintained at RT, clearance techniques and FLPI showed that CFA-treated joints exhibited lower blood flow than saline-treated joints. In cold-exposed animals, this reduction in blood flow disappears, and increased blood flow in the CFA-treated joint is observed using FLPI. Cold-induced increased blood flow in CFA-treated joints was blocked by HC-030031 and not observed in TRPA1 KOs. Cold exposure increased TRPA1 mRNA levels in patellar cartilage, whilst reducing it in synovial membranes from CFA-treated joints.

**Conclusions:**

We provide evidence that environmental cold exposure enhances pain and increases blood flow in a mono-arthritis model. These changes are dependent on TRPA1. Thus, TRPA1 may act locally within the joint to influence blood flow via sensory nerves, in addition to its established nociceptive actions.

## Background

Anecdotal evidence of a link between weather changes and arthritis symptoms has abounded for centuries; several studies have shown that the vast majority of arthritic patients believe their condition to be weather-sensitive [1, 2]. In rheumatoid arthritis, low temperatures in particular are thought to be associated with exacerbated pain [2–4], though this association is contested [5]. One particular issue in trying to delineate this phenomenon is that it is often difficult to distinguish the effect of cold independently from other weather variables, such as pressure and humidity, or indeed the psychological effect of inclement weather [5].

To date, there have been few studies to assess the effect of cold on pain in arthritis. Rats with complete Freund’s adjuvant (CFA)-induced arthritis have an exaggerated hyperalgesic response and sensitized primary afferents after exposure to cold [6, 7], though the underlying mechanisms are not fully understood. One potential mediator is the transient receptor potential ankyrin 1 (TRPA1) channel, shown to be essential for acute cold hypersensitivity in hindpaw inflammatory models [8–10]. TRPA1 is a ligand-gated non-selective Ca^2+^ transducer expressed on neuronal and non-neuronal cells [11, 12]. The channel is activated by temperatures ranging from 10 °C to 17 °C [12, 13], though its role as a cold sensor in vivo is controversial [14]. Conversely, TRPA1 has well-established roles in inflammatory pain, and can be activated by a wide range of endogenous reactive compounds generated by oxidative stress, including hydrogen peroxide [15]. We have previously demonstrated that TRPA1 mediates tumour necrosis factor alpha (TNF-α)-induced inflammatory pain by modulating mechanical hyperalgesia via both the central and peripheral nervous systems [16]. In addition, TRPA1 plays an important role in noxious mechanosensation in normal, inflamed and osteoarthritic models [17], and we have observed a sustained mechanical hyperalgesia in wild-type (WT) but not TRPA1 knockout (KO) mice with CFA-induced mono-arthritis [16].

As well as sensing noxious stimuli, leading to the perception of pain, another key function of sensory nerves is their ability to release potent vasoactive neuropeptides, including substance P (SP) and calcitonin gene-related peptide (CGRP), into the surrounding tissue, leading to neurogenic inflammation [18]. We have shown that TRPA1 activation leads to neuropeptide-dependent vasodilatation [19]. Arthritic patients exhibit alterations in the function of their microvasculature [20], and decreased blood flow in the synovial joint has been observed in rat models of CFA-induced arthritis [21–23]. While the link between environmental cold and blood flow in arthritic joints is not clear at present, rheumatoid arthritis has been associated with Raynaud’s syndrome, which is defined by episodic ischemia of the extremities in response to cold, and defective blood flow regulation [24].

Here, we have established a model of 1 h exposure to 10 °C environmental cold, and used it to investigate the changes in pain sensitivity and blood flow in the knee joint of CFA-induced mono-arthritic mice. Using both TRPA1 antagonists and KO mice, we have investigated whether TRPA1 is linked to these changes. Our results reveal that TRPA1 influences vascular responsiveness and pain sensitivity following cold exposure.

## Methods

### Animals

TRPA1 transgenic mice, as described by [25] were produced using heterozygous breeding pairs (on a mixed genetic background: C57BL/6 J and B6129PF2/J strains) to give litters of mixed genotypes, including TRPA1 KO animals (lacking functional TRPA1), WT control animals (with functional TRPA1), and heterozygous animals. Age-matched male KO and WT mice (25–35 g, 10–12 weeks of age) were used for experiments. For antagonist studies, male CD1 mice (25–35 g, 10–12 weeks of age; Charles River, Margate, UK) were also used. Mice were maintained in a climatically controlled environment (19–21 °C), with access to food and water ad libitum. All experiments were carried out in accordance with the 1986 UK Home Office Animals (Scientific Procedures) Act, with ethical approval from King’s College London. All recovery procedures were performed using 2 % isoflurane anesthesia; 30-gauge needles were used for intra-articular (i.art.) injections (BD Micro-Fine insulin syringes, 0.3 ml; BD Medical, Oxford, UK).

### CFA-induced knee joint inflammation and environmental cold exposure

Mono-arthritis was induced in mice by i.art. injections of CFA (10 μg/10 μl) into the ipsilateral knee joint, and 10 μl saline (0.9 % sodium chloride, pyrogen-free; Baxter Healthcare, Newbury, UK) into the contralateral joint; as in [16]. Inflammation was allowed to develop for 2 weeks, at which point hyperalgesia and knee joint blood flow were assessed. Prior to measurements, animals were maintained at either environmental room temperature (RT; 22–23 °C) or exposed to cold (10 °C) for 1 h. For environmental cold exposure, mice were individually caged with minimal bedding in an enclosed cooled environment with access to food and water. RT humidity was 40.3 ± 1.3 % and cold humidity was 41.7 ± 2.3 %. RT pressure was 1013 ± 1.2 mb and cold pressure was 1013 ± 5.6 mb.

### Nociceptive and weight-bearing experiments

Nociceptive experiments were carried out on 26 CD1, 6 TRPA1 WT and 6 TRPA1 KO mice. For the primary mechanical hyperalgesia measurements, a pressure application measurement device (Ugo Basile, Comerio, Italy; [26]) was used. Mice were scruffed and a digital touch stimulator was placed on the side of the knee joint. An increasing force (at a rate of 30 g/s, as optimized in [26]) was applied to the knee joint with a cut-off of 550 g, and knee joint withdrawal and/or vocalization were used as indicators of nociceptive threshold. Mice were trained once before baseline measurements to prevent freezing during the scruffing procedure. Measurements were taken twice for each joint over a period of 2 days to reduce stress. Results are expressed as knee withdrawal threshold (g). For the measurement of secondary mechanical hyperalgesia, a dynamic plantar aesthesiometer (Ugo Basile) was used, as in [16]. Mice were placed in transparent cages with a wire mesh floor and acclimatized for 30 min before readings started. Measurements were obtained with a straight metal filament (0.5-mm diameter), which exerts an increasing upward force (1 g every 0.1 s) when touching the plantar surface of the hind paw. Measurements were stopped when the paw was withdrawn, and results are expressed as mean paw withdrawal threshold (g) over three experiments. The cut-off force was set at 50 g. Secondary thermal hyperalgesia thresholds were evaluated using the Hargreaves technique (Ugo Basile; as described previously [27]). Mice were placed in behavioural boxes on a glass platform and acclimatized for 30 min. An automatic heat source (50 W, 10 V) was directed at the mouse footpad until paw withdrawal, foot drumming, licking or any other aversive action was observed. A cut-off time of 22 s was used to avoid tissue damage. Measurements were taken in triplicate and mean paw withdrawal latency (s) was calculated.

Weight bearing was determined using an incapacitance tester (Linton Instrumentation, Diss, UK; [28]). Measurements were taken when mice were positioned with their head and front paws resting on the sloping side of the plastic box and hind paws were centred on each force plate with no overlap. Three measurements were taken, and results are expressed as percentage weight on ipsilateral limb. A result of 50 % indicates equal distribution, while values <50 % indicate a preference for weight bearing on the contralateral limb.

All pain behaviour measurements were taken before (baseline) and 2 weeks after mono-arthritis induction. Immediately before the 2-week measurements, mice were either maintained at RT or exposed to cold (10 °C) for 1 h. Experiments were performed in a blinded fashion where possible.

### Knee joint blood flow measurements

Knee joint blood flow was assessed by full-field laser perfusion imager (FLPI; Moor Instruments, Axminster, UK) in 6 WT, 7 TRPA1 KO and 79 CD1 mice. Briefly, 2 weeks after CFA injection, animals were either maintained at RT or exposed to cold (10 °C) for 1 h. Immediately thereafter, mice were fully anaesthetized with a mixture of ketamine (75 mg/kg) and medetomidine (1 mg/kg). Naïve RT-exposed (*n* = 5) and naïve cold-exposed (*n* = 5) CD1 mice were used as controls. Skin overlying the knee joints was removed and the patellar ligament carefully dissected to expose the synovial membrane. Blood flow was recorded in a defined region of interest encompassing the synovial membrane for 30 min. The imager uses speckle contrast imaging to detect changes in blood flow [29, 30]. An arbitrary flux value is produced, which gives the average flow over the defined region of interest. Increases in flux values correspond to increases in blood flow in the joint. Results are expressed as mean flux value for the 30-min recording period.

### ^99m^Tc clearance technique to measure changes in blood flow

Knee joint blood flow was also assessed using a clearance technique [30] in 24 CD1 mice. Naïve RT-exposed (*n* = 5) and naïve cold-exposed CD1 (*n* = 5) mice were used as controls. Two weeks after CFA injection, animals were either maintained at RT or exposed to cold (10 °C) for 1 h, and were then immediately fully anaesthetized with a mixture of ketamine (75 mg/kg) and medetomidine (1 mg/kg). A 20 μl injection of radioactive ^99m^Technetium (^99m^Tc, diluted in saline, approximately 5 kBq per site; Nuclear Medicine, Guy’s Hospital, London) was administered into both knee joints. Immediately after each injection, the radioactivity in the joint was determined using a collimated gamma probe (Europrobe, Bright Technologies, Sheffield, UK). The head of the probe was held against the joint region and counts/min detected in the joint were recorded. Readings were repeated every minute for each joint over a period of 10 min. An equal amount of saline + ^99m^Tc was used as a measurement of total radioactivity, and was measured before and after each experiment to confirm the stability of the radioisotope.

### Real-time PCR analysis

Real-time quantitative PCR [31] was used to assess the effect of inflammation and cold exposure on TRPA1 mRNA expression. Both the contralateral (saline-injected) and ipsilateral (CFA-injected) synovial membrane, patellar cartilage and lumbar L2-L5 dorsal root ganglia (DRG) samples were isolated from arthritic mice exposed to RT or cold, for quantification of TRPA1, TAC-1 (the gene encoding SP) and αCGRP mRNA levels. Samples were collected after blood flow recording, and stored in RNAlater® until RNA extraction was performed. DNA-free total RNA was extracted from samples using the RNeasy Microarray kit (Qiagen, Manchester, UK), and 0.5 μg of total DRG RNA, or 0.15 μg of total synovial membrane and patellar cartilage RNA was reverse transcribed to cDNA using the High Capacity RNA-to-cDNA kit with RNAse inhibitor (Applied Biosystems, Warrington, UK) according to the manufacturer’s instructions. Real-time PCR was performed on a Corbett Rotorgene (hold: 10 min at 95 °C; cycling: 45 cycles of 10 s at 95 °C, 15 s at 57 °C and 5 s at 72 °C; melt: 68–90 °C), using the SensiMix™ SYBR No-ROX Kit (Bioline, London, UK). The following primers were obtained from Sigma-Aldrich, Gillingham, UK:

TRPA1 [forward: 5′-AGGTGATTTTTAAAACATTGCTGAG-3′; reverse: 5′-CTCGATAATTGATGTCTCCTAGCAT-3′], TAC-1 [forward: 5′-AAGCCTCAGCAGTTCTTTGG-3′; reverse: 5′-TCTGGCCATGTCCATAAAGA-3′], αCGRP [forward: 5′-AGCAGGAGGAAGAGCAGGA-3′; reverse: 5′-CAGATTCCCACACCGCTTAG-3′], B_2_M [forward: 5′-CCTGCAGAGTTAAGCATGCC-3′; reverse: 5′-GATGCTTGATCACATGTCTCG-3′], HPRT [forward: 5′-TCCTCCTCAGACCGCTTTT-3′; reverse: 5′-CCTGGTTCATCATCGCTAATC-3′], GAPDH [forward: 5′-GGTCATCCCAGAGCTGAACG-3′; reverse: 5′-TTGCTGTTGAAGTCGCAGGA-3′].

Results are expressed as copy numbers/μl of cDNA, derived using the standard curve method [31]. Data were then plotted against this standard curve using the Rotorgene 6000 series software and expressed as copies/μl of cDNA. Results were normalized to the endogenous housekeeping reference genes (B_2_M, HPRT and GAPDH) using GeNorm (Version 3.4). The geometric mean of the reference genes were calculated using GeNorm and results were normalized by dividing the copies/μl of the target gene(s) of interest by a normalization factor derived by GeNorm [32]. All experiments were performed in accordance with MIQE guidelines [31].

### Pharmacological treatment protocols

To assess the involvement of TRPA1 activation, the selective TRPA1 antagonist HC-030031 (100 mg/kg; Tocris Bioscience, Bristol, UK) or vehicle (10 % DMSO in saline; *n* = 7) was administered intraperitoneally (i.p.) to CD1 mice 30 min prior to cold exposure [30]. The contribution of CGRP to blood flow was analysed by pretreatment with the CGRP antagonist CGRP_8–37_ (400 nmol/kg; Tocris Bioscience), administered intravenously (i.v.) 15 min prior to recordings [33]. The participation of SP was evaluated by systemic administration of neurokinin 1 (NK_1_) receptor antagonist SR140333 (480 nmol/kg; i.v.; Sigma-Aldrich) 15 min prior to blood flow measurements [33]. The vehicle (2 % DMSO in saline) was the same for both CGRP_8–37_ and SR140333.

### Statistical analysis

Results are expressed as mean ± SEM from *n* number of animals. Statistical analyses were performed by two-way analysis of variance (ANOVA) or repeated measures two-way ANOVA, as appropriate, followed by Bonferroni’s adjustment for multiple comparisons. *p* values < 0.05 were considered significant.

## Results

### Cold exposure causes bilateral pain sensitivity in knee joints of mice with CFA-induced mono-arthritis, in a TRPA1-dependent manner

**Fig. 1:**
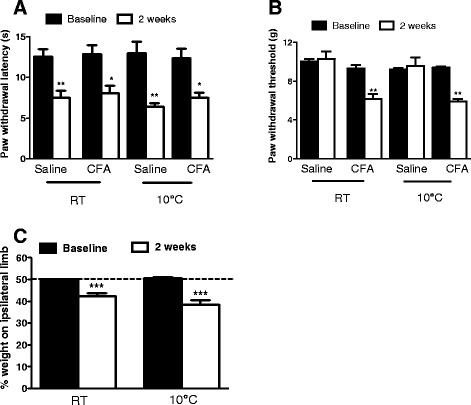
Nociceptive thresholds and weight-bearing distribution in mice with CFA-induced mono-arthritis maintained either at RT or exposed to cold (10 °C) for 1 h prior to measurements. **a** Secondary thermal hyperalgesia (using Hargreaves test). **b** Secondary mechanical hyperalgesia (using dynamic plantar aesthesiometer). **c** Weight bearing (using incapacitance tester). Measurements were taken before (baseline) and 2 weeks after mono-arthritis induction. *n* = 6 ^*^ *p* < 0.05, ^**^ *p* < 0.01, ^***^ *p* < 0.001 compared to baseline, using repeated measures two-way ANOVA and Bonferroni’s multiple comparisons. *CFA* complete Freund’s adjuvant, *RT* room temperature

The effect of environmental cold on the pain threshold of arthritic mice was evaluated through a range of nociceptive parameters, namely the measurement of primary and secondary mechanical hyperalgesia, thermal hyperalgesia and weight-bearing distribution (Fig. 1). No differences in baseline measurements were observed between the groups. Two weeks after i.art. CFA injection, unilateral secondary mechanical hyperalgesia and bilateral secondary thermal hyperalgesia were observed both in mice maintained at RT and mice exposed to cold (Fig. 1a and b), thus cold exposure had no effect on secondary measures of pain.

The incapacitance test was used to evaluate static weight bearing in unrestrained mice, thus allowing for quantification of pain-related guarding behaviour. At 2 weeks post-injection, mice were placing significantly less weight through the ipsilateral CFA-treated hindlimb compared to baseline. The effect was observed both in mice maintained at RT and mice exposed to cold (Fig. 1c).

**Fig. 2:**
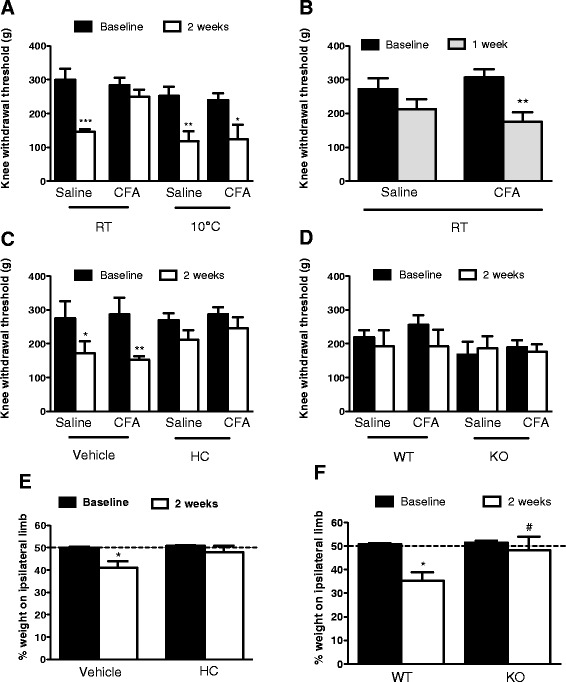
Role of TRPA1 in pain behaviour of CFA-induced mono-arthritic mice after cold exposure. **a** Primary mechanical hyperalgesia (measured using pressure application measurement) before and 2 weeks after mono-arthritis induction in mice maintained at RT or exposed to cold (10 °C) for 1 h prior to measurements. **b** Primary mechanical hyperalgesia before and 1 week after mono-arthritis induction in mice maintained at RT. Primary mechanical hyperalgesia in (**c**) CD1 mice pretreated with vehicle or HC-030031 and (**d**) WT and TRPA1 KO mice 2 weeks after CFA injection and following 1 h cold exposure, *n* = 6–8, ^*^ *p* < 0.05, ^**^ *p* < 0.01 compared to baseline, using repeated measures two-way ANOVA and Bonferroni’s multiple comparisons. Weight bearing in (**e**) mice pretreated with vehicle or HC-030031 and (**f**) WT and TRPA1 KO mice 2 weeks after CFA injection and following 1 h cold exposure, *n* = 6–13, ^*^ *p* < 0.05 compared to baseline, ^#^ *p* < 0.05 compared to WT, using one-way ANOVA and Bonferroni’s multiple comparisons. *CFA* complete Freund’s adjuvant, *KO* knockout, *RT* room temperature, *TRPA1* transient receptor potential ankyrin 1, *WT* wild-type

A pressure application measurement device was used to directly test primary mechanical hyperalgesic thresholds of the knee joint. With this technique, mice exposed to 1 h of cold (10 °C) exhibited significant decreases in withdrawal thresholds in both ipsilateral and contralateral joints (Fig. 2a), suggesting that cold exposure had increased pain sensitivity in both knee joints. Interestingly, mice maintained at RT did not exhibit a decrease in withdrawal threshold from baseline in the CFA-treated joint, but did show a significant decrease from baseline in the contralateral saline-treated joint (Fig. 2a). Different time courses between ipsilateral and contralateral pain have often been reported [16, 34, 35]. Thus, in our study, the hyperalgesia observed in the CFA-treated knee joint of animals exposed to RT may have resolved faster than the contralateral hyperalgesia. This was tested by examining the knee withdrawal thresholds 1 week post-CFA and -saline treatment. At this time point, mice maintained at RT had a significant decrease in threshold in the CFA-treated joint but not in the saline-treated joint (Fig. 2b). Cold-exposed mice had no differences in thresholds in either joint (Fig. 2b), suggesting that at this early time point in the progression of arthritis, cold may have an analgesic effect.

We investigated the role of TRPA1 in the cold-induced bilateral hyperalgesia at 2 weeks post-CFA injection. Vehicle-treated CD1 mice exhibited significant reductions in ipsilateral and contralateral knee withdrawal thresholds 2 weeks after i.art. CFA and 1 h exposure to cold (Fig. 2c), indicative of contralateral as well as ipsilateral hyperalgesia. However, pretreatment with the TRPA1 antagonist, HC-030031, prevented any significant decreases in knee withdrawal thresholds 2 weeks after i.art CFA in cold-exposed animals (Fig. 2c). HC-030031 also blocked the significant decrease in weight bearing through the CFA-treated hindlimb at the same time point (Fig. 2e). TRPA1 KO mice also did not exhibit any changes in knee withdrawal threshold (Fig. 2d). Surprisingly, TRPA1 WT mice did not show significant changes in their knee withdrawal thresholds (Fig. 2d), however WT mice did place significantly less weight through the CFA-treated hindlimb at 2 weeks than at baseline (Fig. 2f). TRPA1 KO mice did not show any significant changes in knee withdrawal thresholds (Fig. 2d), and no changes in weight bearing were observed (Fig. 2f).

### Knee joint blood flow differs between RT and cold-exposed mice

**Fig. 3:**
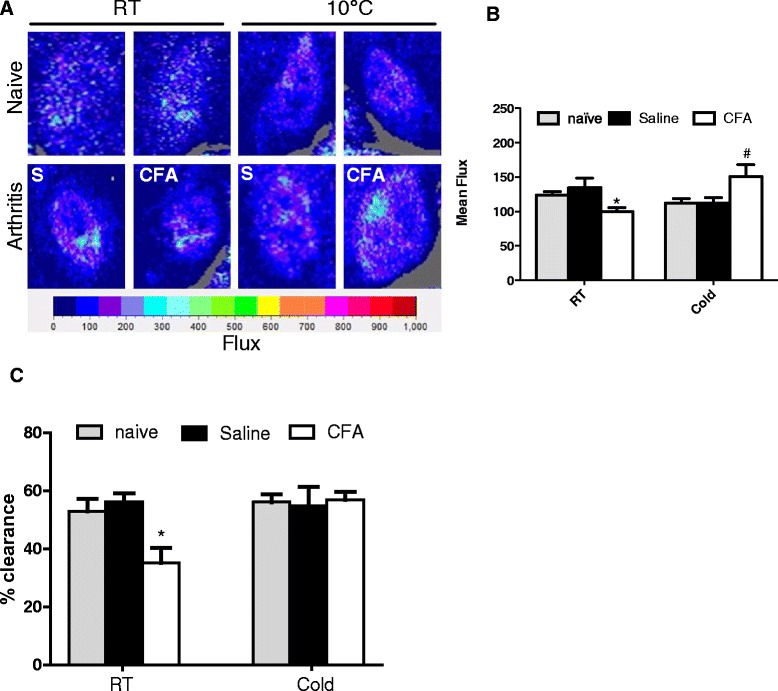
Effect of cold exposure on knee joint blood flow in animals with CFA-induced mono-arthritis. Mono-arthritis was induced in animals by injection of CFA (ipsilateral joint) and saline (contralateral joint). **a** FLPI images of blood flow in the recorded regions of interest from the synovial membrane in naïve and arthritic animals following 1 h exposure to RT or cold (10 °C) 2 weeks after CFA injection. **b** Average blood flow measured using FLPI in joints of naïve and arthritic animals at RT or exposed to cold, *n* = 7–8, ^*^ *p* < 0.05 compared to cold-exposed CFA-treated joint, ^#^ *p* < 0.05 compared to cold-exposed naïve and saline-treated joints. **c** Percentage clearance of i.art. ^99m^Technetium in the knee joint after 5 min in arthritic animals at RT or exposed to cold, *n* = 7, ^*^ *p* < 0.05 compared with saline-treated counterparts. *CFA* complete Freund’s adjuvant, *FLPI* full-field laser perfusion imager, *RT* room temperature

Experiments using the FLPI showed that CD1 mice maintained at RT exhibited reduced blood flow in their CFA-treated joint (Fig. 3a and b). Cold exposure caused a significant increase in flux in the synovial membrane of ipsilateral CFA-treated joints compared to contralateral saline-treated joints, naïve controls, and RT-maintained CFA-treated joints (Fig. 3a and b). This increase in flux is indicative of increased blood flow in the synovial membrane after exposure to cold in the CFA-treated joint. Experiments using the ^99m^Tc clearance technique showed that in RT-maintained mice, the CFA-treated joint cleared ^99m^Tc significantly slower over the whole time course than the control saline-treated joint (Fig. 3c shows percentage clearance after 5 min, at which point naïve mice exhibit approximately 50 % clearance). This difference in clearance disappeared when mice were exposed to cold for 1 h (Fig. 3c).

### TRPA1 mediates the cold-induced increase in synovial blood flow in arthritic mice

**Fig. 4:**
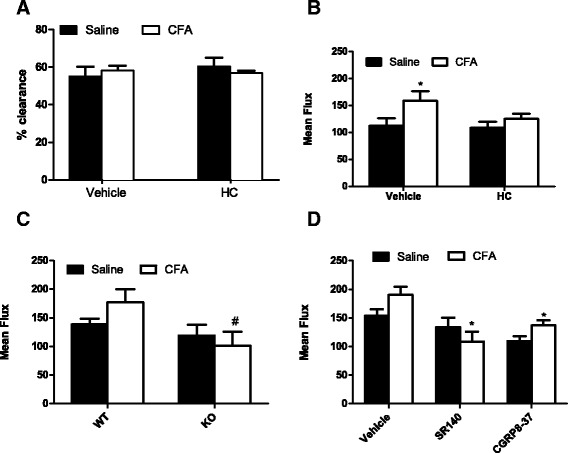
Participation of TRPA1, SP and CGRP in cold-induced increases in blood flow in arthritic animals. **a** Percentage clearance of i.art. ^99m^Technetium in the knee joint after 5 min in arthritic animals pretreated with selective TRPA1 antagonist, HC-030031 (100 mg/kg; i.p.), and exposed to cold (10 °C) for 1 h, *n* = 6. **b** Knee joint blood flow recorded using FLPI from arthritic animals pretreated with selective TRPA1 antagonist, HC-030031 (100 mg/kg; i.p.; 1 h), and exposed to cold (10 °C) for 1 h, *n* = 6–7, ^*^ *p* < 0.05 compared to vehicle-treated contralateral joint. **c** Knee joint blood flow recorded using FLPI from arthritic WT and TRPA1 KO mice exposed to cold (10 °C) for 1 h, *n* = 6–7, ^#^ *p* < 0.05 compared to WT CFA joint. **d** Knee joint blood flow recorded using FLPI in arthritic animals pretreated systemically with the selective NK_1_ antagonist SR140333 (480 mg/kg; i.v.; 15 min, *n* = 7) or the CGRP antagonist CGRP_8–37_ (400 mg/kg; i.v.; 15 min, *n* = 7), and exposed to cold (10 °C) for 1 h. For the vehicle-treated group, *n* = 13, ^*^ *p* < 0.05 compared to vehicle-treated ipsilateral joint. *CFA* complete Freund’s adjuvant, *CGRP* calcitonin gene-related peptide, *FLPI* full-field laser perfusion imager, *KO* knockout, *NK*_*1*_ neurokinin 1, *SP* substance P, *TRPA1* transient receptor potential ankyrin 1, *WT* wild-type

We investigated the role of TRPA1 in mediating the differences in blood flow caused by cold exposure in animals with 2-week mono-arthritis. Figure 4a shows that pretreatment with the selective TRPA1 antagonist HC-030031 (100 mg/kg; i.p.) before the 1 h cold exposure had no effect on ^99m^Tc clearance. However, HC-030031 blocked the increase in synovial blood flow observed using FLPI after cold exposure (Fig. 4b). TRPA1 WT mice had increased synovial blood flow in their CFA-treated joint compared to saline-treated joint, though this did not reach significance (Fig. 4c). Following cold exposure, TRPA1 KO mice exhibited significantly reduced blood flow in their CFA-treated joints compared to their WT counterparts (Fig. 4c).

### CGRP and SP are involved in the cold-induced increase in synovial blood flow in arthritic mice

We next investigated the contribution of neuropeptides to the cold-induced changes in knee joint blood flow in animals with 2-week mono-arthritis caused by CFA. Both SP and CGRP are known to mediate inflammation and pain sensation in arthritic patients and animal models of arthritis [36–39], and to be released from sensory neurons following activation of TRPA1 receptors [40, 41]. Correspondingly, systemic treatment with either the selective NK_1_ antagonist SR140333 (480 nmol/kg; i.v.; 15 min) or the selective CGRP antagonist CGRP_8–37_ (400 nmol/kg; i.v.; 15 min) prevented the increase in blood flow after cold exposure (Fig. 4c).

### Cold exposure alters TRPA1, TAC-1 and αCGRP mRNA expression in knee joint tissues following CFA injection

Table 1 shows TRPA1 mRNA expression in DRG, patellar cartilage and synovial membrane samples obtained from the contralateral (saline-injected) and ipsilateral (CFA-injected) sides 2 weeks after mono-arthritis induction. No significant differences in TRPA1 mRNA expression were found between contralateral and ipsilateral sides in any samples obtained from RT-exposed animals (Table 1). Cold exposure did not affect TRPA1 mRNA expression in DRG samples collected from either the contralateral or the ipsilateral sides (Table 1). Interestingly, cold exposure caused a significant downregulation of TRPA1 mRNA in synovial membrane samples, whilst a marked upregulation of TRPA1 mRNA was evident in patellar cartilage samples collected from the ipsilateral CFA-injected side (Table 1).

**Table 1:** mRNA expression in arthritic mice exposed to cold

Gene	Tissue	RT		10 °C	
		Saline	CFA	Saline	CFA
*TRPA1*	DRG	862.76 ± 62.84	1116.79 ± 109.20	1005.35 ± 98.58	879.87 ± 63.95
*TRPA1*	Synovial membrane	24.17 ± 5.48	20.09 ± 5.41	31.98 ± 6.98	7.69 ± 0.98^*^
*TRPA1*	Patellar cartilage	41.20 ± 8.21	48.94 ± 10.15	45.56 ± 8.14	115.00 ± 42.37^*#^
*TAC-1*	DRG	8939.63 ± 1547.03	10197.42 ± 911.50	11325.64 ± 1143.52	7620.78 ± 1708.66
*TAC-1*	Synovial membrane	346.28 ± 70.11	132.70 ± 16.78^*^	362.31 ± 72.70	249.36 ± 99.34
*TAC-1*	Patellar cartilage	145.37 ± 42.49	86.91 ± 25.93	89.69 ± 24.76	214.74 ± 71.01
*CGRP*	DRG	309931.4 ± 38430.2	370243.6 ± 45187.4	391659.9 ± 35192.5	252244.4 ± 52594.3
*CGRP*	Synovial membrane	68.73 ± 15.55	48.38 ± 18.91	57.44 ± 6.89	24.98 ± 1.42
*CGRP*	Patellar cartilage	43.38 ± 8.47	32.64 ± 9.02	33.98 ± 7.48	120.63 ± 46.56^*#^

TRPA1, TAC-1 and CGRP mRNA expression in DRG, synovial membrane, and patellar cartilage samples obtained from RT-and cold-exposed mice with 2-week CFA-induced monoarthritis. Data are represented as copy numbers per μl of pure cDNA normalized by comparison to B_2_M, HPRT and GAPDH in contralateral (saline-injected) and ipsilateral (CFA-injected) sides, *n* = 4–17, ^*^ *p* < 0.05, relative to saline-treated counterparts, ^#^ *p* < 0.05 relative to RT-exposed CFA-treated counterparts

*RT* room temperature, *CFA* complete Freund’s adjuvant, *DRG* dorsal root ganglia

DRG mRNA expression levels of both αCGRP and TAC-1 were similar between ipsilateral and contralateral sides, irrespective of temperature (Table 1). There was a significant reduction in TAC-1 expression in ipsilateral CFA-treated synovial membrane samples from mice maintained at RT. This reduction was lost in mice exposed to cold (Table 1). Similar to TRPA1 expression changes, αCGRP expression levels were upregulated in ipsilateral CFA-treated patellar cartilage samples from cold-exposed mice (Table 1).

## Discussion

It is not known by what mechanism environmental cold affects arthritis sufferers, though it is often reported as an exacerbating factor for their condition. In this study, we have used the CFA-induced mono-arthritis model with exposure to cold (10 °C for 1 h), to investigate the effect of acute environmental cold exposure on symptoms. We show for the first time that after this acute exposure, bilateral pain sensitivity occurs in the knee joints of cold-exposed arthritic mice 2 weeks after induction of arthritis, and that this is dependent on the cold sensor, TRPA1. Importantly, we also reveal that increased blood flow is seen in the CFA-treated knee joint after cold exposure. This is also dependent on TRPA1, and involves the vasodilatory neuropeptides, SP and CGRP.

We used a range of behavioural pain measurements in our model. Secondary mechanical hyperalgesia was observed after induction of CFA-induced mono-arthritis, and was similar in both RT- and cold-exposed animals, in keeping with previously published reports [16, 34]. Bilateral thermal hyperalgesia was detected 2 weeks after unilateral CFA injection, irrespective of temperature. Contralateral effects during arthritis are commonly seen both in animal models and humans [35, 42, 43] so this was not a surprising result. Interestingly, using the pressure application measurement technique to record primary mechanical hyperalgesia of the knee joint, we have shown that mice maintained at RT had a significant decrease in threshold only in their contralateral saline-treated joint and not in the ipsilateral CFA-treated joint 2 weeks after arthritis induction. Mechanisms underlying ipsilateral and contralateral pain differ [27, 44], with the latter thought to arise from central neurogenic mechanisms [42, 45]. After induction of unilateral arthritis, spontaneous antidromic action potentials have been recorded in contralateral sensory nerves [42, 45] and contralateral inflammatory cytokine generation has been observed [27]. One week after arthritis induction, mice maintained at RT exhibited pain sensitivity in the CFA-treated joint but not in the saline-treated joint (Fig. 2b). Different time courses between ipsilateral and contralateral pain have commonly been reported [16, 34, 35], with resolution of ipsilateral pain occurring as the pain on the contralateral side starts to manifest. We have previously shown in a model of TNF-α-induced mechanical hypersensitivity that peripheral and central TRPA1 channels are important at different time points during the development of ipsilateral and contralateral pain hypersensitivity [16]. Importantly, the cold-exposed animals exhibited hyperalgesia in both joints at 2 weeks, indicative of cold-induced pain exacerbation.

We have previously demonstrated a role for TRPA1 in the secondary mechanical hyperalgesia associated with CFA-induced mono-arthritis [16], but until now no study has assessed a role for TRPA1 in arthritic pain exacerbation caused by environmental cold. Though there is still discussion over whether and how TRPA1 can act as a cold sensor for nociceptive responses in vivo [14], there is much evidence linking TRPA1 to cold hypersensitivity arising during inflammatory or neuropathic conditions [8–10, 46]. We provide evidence that the bilateral pain in arthritic animals exposed to cold is TRPA1-dependent, as no significant hyperalgesia, when measured with the pressure application measurement device, was observed in both contralateral and ipsilateral knee joints of mice treated with HC-030031 (Fig. 2c). Surprisingly, TRPA1 WT mice did not show any threshold differences between saline- and CFA-treated joints. The TRPA1 genetically modified mice were generated on a mixed genetic background containing both C57BL/6 J and B6129PF2/J strains. Different mouse strains are known to have differing nociceptive sensitivities [16, 47] and several groups have reported issues with detecting pain differences specifically in C57BL/6 mice [48, 49]. The variability in pain thresholds was greater in TRPA1 WT mice than KO mice 2 weeks after arthritis induction, and may be indicative of increased stress from the handling and restraint necessary for pressure application measurement. Despite the problems with the TRPA1 WT mice, data using the TRPA1 antagonist suggests TRPA1 does play a role in the bilateral pain observed after cold exposure. It is possible that TRPA1 is not directly activated by cold in this study. Our studies in skin suggest that TRPA1 very quickly senses noxious cold [30], however, here we have a longer time course which may facilitate lipid metabolism. It is well established that cold exposure activates the sympathetic nervous system resulting in liberation of free fatty acids (FFA) and glycerol, from triglyceride hydrolysis in adipose tissue, which are then released in the circulation and mobilized for energy [50]. However, we are not aware of possible interactions between FFA and/or glycerol with TRPA1, although, we have previously shown an interaction with TRPA1 and the sympathetic nervous system’s principal neurotransmitter, noradrenaline [30]. Therefore, it is likely that the results observed in this study are upstream of lipid metabolism.

Vascular remodelling in the joint is a common feature of arthritis, with the growth of blood vessels from the subchondral bone into articular cartilage [51]. Sensory nerve growth occurs together with the angiogenesis, linking vascular effects to the development of joint pain [39, 51, 52]. We used two different techniques to examine blood flow in the knee joints of mono-arthritic animals maintained at RT or exposed to cold. FLPI allows real-time imaging of blood flow specifically in the synovial membrane of the knee joint, whereas clearance of i.art. injection of ^99m^Technetium is a measure of total blood flow in the entire joint. Both techniques showed a reduction in blood flow in CFA-treated joints compared to contralateral saline-treated joints in animals maintained at RT, in keeping with previous reports [21–23]. This is also consistent with clinical observations of low microcirculatory flow in arthritic joints of patients with rheumatoid arthritis, and is thought to be due to increased microvascular resistance, as a result of inflammation-induced dysfunction [20].

The primary objective of this study was to investigate the effects of environmental cold on arthritis and its associated mechanisms. In contrast to animals maintained at RT, the CFA-treated joint of cold-exposed animals did not exhibit reduced blood flow compared to the saline-treated joint as shown using the FLPI and ^99m^Technetium clearance techniques. Indeed, using FLPI, we recorded increased blood flow in the CFA-treated joint compared to saline-treated joint in cold-exposed CD1 mice. FLPI specifically detects changes in blood flow in the synovial membrane by illuminating the region with a laser and recording speckle patterns produced by moving red blood cells, thus generating flux values correlating to real-time blood flow [29]. Therefore, this is a more sensitive method of quantifying blood flow than ^99m^Technetium clearance, which measures the rate of clearance of ^99m^Technetium from the intra-articular cavity into the blood stream, and can be affected by various factors [53]. Indeed, no changes in clearance rates were observed after pretreatment with HC-030031 in cold-exposed animals, however with FLPI, pretreatment with HC-030031 prevented the increase in blood flow seen after cold exposure in vehicle-treated animals, suggesting the involvement of TRPA1. In addition, TRPA1 KO mice have significantly reduced blood flow in their CFA-treated joints compared to their WT counterparts.

As yet, the functional importance of these changes in blood flow after cold is not clear, so it is not known whether these blood flow changes would be detrimental or beneficial to the arthritic joint. However, as well as altering blood flow, we have shown that TRPA1 blockade reduces joint pain, and this would be a positive effect.

We have previously shown that activation of TRPA1 causes vasodilatation that is neuropeptide-dependent [19]. Thus, we examined a role for SP and CGRP in the cold-induced increase in blood flow. Systemic administration of both the SP NK_1_ receptor antagonist, SR140333, and the CGRP antagonist, CGRP_8–37_, prevented the increase in blood flow observed in CFA-treated joints after cold exposure. However, the antagonists also decreased blood flow in the contralateral saline-treated joints, suggesting that SP and CGRP are involved in general blood flow changes after cold, independently of inflammation.

We examined mRNA expression levels of TRPA1, TAC-1 and CGRP from joint tissues in mice maintained at RT or exposed to cold, 2 weeks after arthritis induction, in order to detect the start of any potential changes caused by acute cold exposure. A recent study has shown that expression of the growth factor, vascular endothelial growth factor (VEGF), and the inflammatory mediator, interleukin-1 (IL-1), are increased in cartilage cells from rats with CFA-induced arthritis after exposure to low temperatures [54]. TRPA1, TAC-1 and CGRP are all highly expressed in DRGs, and we observed no significant differences in their expression under any of the treatment conditions. The only gene altered by CFA treatment in RT-exposed animals was TAC-1, which was significantly reduced in the synovial membrane of the ipsilateral joint. This is perhaps surprising, given that SP-positive nerve fibres are present in the synovium, and increased sensory neuronal growth is known to occur in arthritic joints [39]. However, other groups have reported a reduction in SP-containing synovial nerves during inflammatory arthritis, suggesting that these nerves may be destroyed by proteolytic enzymes released from inflammatory cells during the course of the disease [55]. TRPA1 has been suggested to be expressed in non-neuronal cells, including vascular cells [11] and synoviocytes [56], although lack of a selective anti-murine TRPA1 antibody has limited studies of protein expression. We found low levels of TRPA1 mRNA expression in the patellar cartilage and synovial membrane in all samples, supporting the concept that TRPA1 gene expression is present in the joint. Whether this expression arises from neuronal or non-neuronal sources, is unclear at this stage. Cold exposure increases the expression of TRPA1 and CGRP mRNA in the patellar cartilage of the CFA-treated joint but decreased levels of TRPA1 mRNA are observed after cold in the synovial membrane from the CFA-treated joint. Vascular remodeling leads to destruction of the synovial lining, and is associated with neuronal growth into the cartilage in arthritic joints [51]. However, it would be surprising if structural changes could account for differences between RT-exposed control joints and joints exposed to cold for just 1 hour. Although we are presently unable to account for the differences in gene expression observed in this study, it is possible that these changes have a direct impact on blood flow and pain sensitivity under cold exposure. Indeed, it has been suggested that cold exposure leads to increased expression of inflammatory mediators in arthritic joints, such as VEGF, a key angiogenic factor, [54]. Hence expression of key inflammatory and vascular factors may represent the initial step towards altered joint pathology, leading to an exacerbation of arthritic symptoms following prolonged exposure to cold. Interestingly, a recent study detected increased levels of TRPA1 expression in the skin of subjects with higher pain thresholds, and this expression was regulated through differential methylation of the TRPA1 promoter [57]. Thus, TRPA1 may contribute to pain sensitivity through tight regulation of its expression.

## Conclusions

This study provides the first evidence that environmental cold exposure alters pain sensitivity and blood flow in a murine model of arthritis. Our findings are in keeping with the concept that pharmacological manipulation of peripheral TRPA1 channels may attenuate changes in blood flow and hyperalgesia associated with arthritis, particularly in cold conditions.

## Abbreviations

^99m^Tc: ^99m^Technetium
CFA: Complete Freund’s adjuvant
CGRP: Calcitonin gene-related peptide
DRG: Dorsal root ganglia
FFA: Free fatty acids
FLPI: Full-field laser perfusion imager
IL-1: Interleukin-1
KO: Knockout
NK1: neurokinin-1
RT: Room temperature
SP: Substance P
TNF: tumour necrosis factor
TRPA1: Transient receptor potential ankyrin 1
VEGF: Vascular endothelial growth factor
WT: Wild-type
